# Nurse roles in antimicrobial stewardship: lessons from public sectors models of acute care service delivery in the United Kingdom

**DOI:** 10.1186/s13756-019-0621-4

**Published:** 2019-10-22

**Authors:** Enrique Castro-Sánchez, Mark Gilchrist, Raheelah Ahmad, Molly Courtenay, Jo Bosanquet, Alison H. Holmes

**Affiliations:** 1 NIHR Health Protection Research Unit (HPRU) in Healthcare Associated Infection (HCAI) and Antimicrobial Resistance (AMR) at Imperial College London, Du Cane Road, London, UK; 2 Imperial College Healthcare NHS Trust, London, UK; 3 Business School, Management Department, Imperial College London, London, UK; 4 School of Health Sciences, Cardiff University, Cardiff, UK; 5 Antimicrobial Resistance Programme, Public Health England, London, UK

**Keywords:** Antimicrobial stewardship, Nursing, Service delivery, Implementation

## Abstract

**Background:**

Health care services must engage all relevant healthcare workers, including nurses, in optimal antimicrobial use to address the global threat of drug-resistant infections. Reflecting upon the variety of antimicrobial stewardship (AMS) nursing models already implemented in the UK could facilitate policymaking and decisions in other settings about context-sensitive, pragmatic nurse roles.

**Methods:**

We describe purposefully selected cases drawn from the UK network of public sector nurses in AMS exploring their characteristics, influence, relations with clinical and financial structures, and role content.

**Results:**

AMS nursing has been deployed in the UK within ‘vertical’, ‘horizontal’ or ‘hybrid’ models. The ‘vertical’ model refers to a novel, often unique consultant-type role ideally suited to transform organisational practice by legitimising nurse participation in antimicrobial decisions. Such organisational improvements may not be straightforward, though, due to scalability issues. The ‘horizontal’ model can foster coordinated efforts to increase optimal AMS behaviours in all nurses around a narrative of patient safety and quality. Such model may be unable to address tensions between the required institutional response to sepsis and the inappropriate use of antibiotics. Finally, the ‘hybrid’ model would increase AMS responsibilities for all nurses whilst allocating some expanded AMS skills to existing teams of specialists such as sepsis or vascular access nurses. This model can generate economies of scale, yet it may be threatened by a lack of clarity about a nurse-relevant vision.

**Conclusions:**

A variety of models articulating the participation of nurses in antimicrobial stewardship efforts have already been implemented in public sector organisations in the UK. The strengths and weaknesses of each model need considering before implementation in other settings and healthcare systems, including precise metrics of success and careful consideration of context-sensitive, resource dependent and pragmatic solutions.

## Background

Responding to the global threat of drug-resistant infections (DRIs) demands a blend of wide-ranging yet integrated technical and organisational approaches. Some of these approaches include clinical practice improvements, education and training, surveillance, research and policy [1].

Such response to DRIs, however, must also overcome some workforce dilemmas and challenges. On the one hand, there have been proposals for governments, healthcare leaders and other relevant actors to increase funds and boost the number of frontline professionals involved in relevant actions against drug resistance [2]. Alas, the current supply of healthcare professionals worldwide is not robust enough to meet such demands, with shortages of healthcare workers estimated to reach ~ 13 million by 2035 [3]. In the UK alone, for example, more than 40,000 nursing positions remain vacant. Therefore, it remains crucial to maximise the contribution of existing professional cadres towards appropriate use of antimicrobials.

Among these cadres, nurses represent the largest workforce worldwide. In India, for example, there were just under 4 million nurses in 2016 [4], compared with the 2.95 million nurses practising in the US [5], or the 287,000 full-time equivalent nurses and health visitors in the UK at the end of 2017 [6]. Arguably, any marginal gain –say, 1%– in the involvement of nurses across optimal management of antibiotics to their full clinical and leadership potential might have a compounded beneficial effect for health services and patients. Such benefits would be particularly welcomed in settings where scarcity of human resources is most acute and the struggle to establish ‘more traditional’ AMS teams (i.e., those formed by a physician and a pharmacist) more pressing.

Endorsements for the integration or leadership of nurses within AMS interventions have already been made [7, 8]; however, the majority of authors and position statements published have sketched such participation primarily around bedside, clinical, assistive and task-oriented antimicrobial management. Those aspects, although relevant and appropriate, still omit nurses from the wider decision-making and executive process and therefore constrain their potential influence and leadership on relevant antimicrobial outcomes [9].

The drivers for such limiting perspective may be multiple, from perceptions within nursing and other healthcare professions about AMS as a mere technical process requiring increased knowledge about antibiotic prescriptions [10] and which therefore broadly excludes professionals without prescribing powers, to existing gaps in undergraduate and postgraduate education about AMS and resistance across human health disciplines [11, 12].

Gradually, though, a variety of interventions such as the standardisation of educational competencies [13, 14] (Table 1**)** and the regulation of professional AMS tasks [15, 16] –both aspects crucial considering that effective and sustainable AMS programs demand coordinated efforts from multiple professional groups– have endeavoured to close those gaps and balance their impact.

**Table 1:** Proposed antimicrobial stewardship education domains for undergraduate students in healthcare disciplines in the United Kingdom, [13]

Domain One: Infection prevention and control	
Domain Two: Antimicrobials and antimicrobial resistance	
Domain Three: The diagnosis of infection and the use of antimicrobials	
Domain Four: Antimicrobial prescribing practice	
Domain Five: Person centred care	
Domain Six: Interprofessional collaborative practice	

**Fig. 1:**
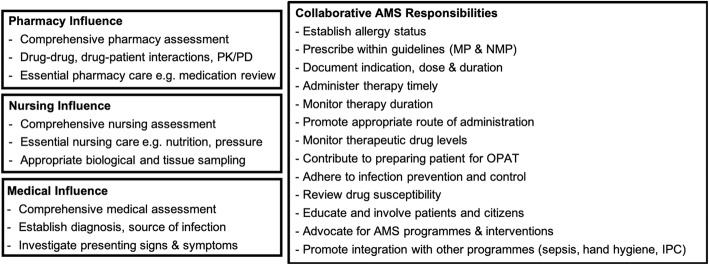
Suggested roles and responsibilities for medical, pharmacy, and nursing staff as part of a stewardship team (adapted from [19])

The maturity now reached by the notion of nurse participation in AMS has also been reflected in some of the new domains emerging for such participation, ranging from leadership [17], political advocacy [18], or funding of services [19]. (Fig. 1**)** This progressive inclusion of nurses onto stewardship therefore presents a good opportunity to reflect upon some of the models of AMS nursing already implemented, with a view to facilitate efforts by healthcare workers and decision-makers considering the introduction or expansion of efforts.

## Methods

The paper uses information from purposefully selected, convenience cases drawn from the nationwide network of public sector nurses in AMS in the UK exploring their salient characteristics, strategic influence, managerial and financial relations with other structures, role content and effect on professional identity. The cases were selected to ensure sufficiently rich and contrasting information on their characteristics was obtained, from London/outside London healthcare services, urban and rural organisations, reference and district hospitals, and role components. The cases would help reflect upon lessons and propose models that could be applicable internationally, thus facilitating the implementation of context-sensitive and pragmatic nurse roles.

The network, akin to a special interest group or a community of practice [20], formed organically following the 1st nursing summit in AMS held at Imperial College London in the UK in January 2017. The network currently includes 109 nurses interested in progressing clinical, academic, managerial and educational aspects of nurse involvement in AMS, establishing mechanisms of support for new entrants to the field, fostering collaborations and ultimately encouraging professional visibility at a time of calls for increased nursing leadership in the area [21]. The network has received robust support from clinical and research leaders from pharmacy and medicine. Although the majority of members are based in the UK, there are also professionals from Sweden, Australia, Norway, South Africa, Nigeria and the Netherlands.

The information provided on enrolment to the network by purposively selected cases was mapped against role specifications, scope of practice, governance and organisational profiles **(**Table 2**)**, with attention to how closely the nurse roles resembled those previously introduced among physicians and pharmacists [22], the setting of jurisdiction and practice for the nurses (community, hospital, acute, long-term care …), the organisational placing of the role (i.e., whether embedded in infection prevention and control or antimicrobial stewardship pharmacy teams), specific role components (i.e., clinical, educational, quality or service improvement, policy-making, managerial), and measures of role process and outcome evaluation (that is, which indicators would deem the roles as successful).

**Table 2:** Domains of antimicrobial stewardship nursing model analysis

Interprofessional working: Considers whether interprofessional working (working with and across professions) is required for the role within the model.	
Strategic influence and relation with other structures, teams or services: Defines the likely influence of the model at strategic and organisational level, as well as the position of role holders in relation to other organisational or professional structures.	
Clinical outcomes: Describes whether changes resulting from introduction of model would reflect impact or process achievements.	
Individual identity: Examines the role archetype used within the antimicrobial stewardship nursing model (i.e. consultant nurse-type, specialist nurse-type, or staff nurse-type).	
Funding/Managerial structures: Describes managerial and supervisory responsibility for nurses, as well as the kind of funding (pilot, short-term, etc. …) and accountability for appointing the workers.	
Setting of practice: Hospital, community, long-term care, nursing home facilities …	
Role components: Includes clinical, educational, quality improvement, policy, and managerial components.	

## Results

Nursing involvement and participation in stewardship has adopted three broad yet distinct models (Table 3), in close alignment with other clinical areas where innovative roles and competencies have appeared [23–26].

**Table 3:** Characteristics of antimicrobial stewardship nursing models

Domains							
Antimicrobial stewardship nursing model	Interprofessional working	Strategic influence- Relation with other structures	Clinical outcomes (What measure of impact? Process?)	Individual identity	Funding/ Managerial structures	Setting of practice (hospital, community …)	Role components (clinical, educational, quality, policy, managerial)
Vertical (i.e. nurse consultant)	Yes	High strategic influence; focal relation with comparable figures/ roles within own profession (i.e. nurse consultant) or others (i.e. pharmacy consultant); collaboration/leadership across aligned areas (i.e. AMS & IPC/AMS & sepsis etc)	May be difficult to robustly attribute impact or clinical improvements to the role in view of indirect work (i.e. influencing others) Feasible to attribute process improvements	Novel professional figure/role, supported by similar professionals in other clinical areas, or professionals from other disciplines	Mainstream human resources funding May be difficult to evaluate value-for-money Appointed by board-level managers from own or other professions	Hospital or community, but most likely hospital	All, with emphasis on planning/ evaluation/ management of organisational practice
Hybrid (i.e. nurse specialist)	Yes	Some strategic influence as part of specialist services; advisory relation with own and other professions across multiple areas	Easy to attribute impact or clinical improvements due to focus on planning and delivery of clinical services, education.	Traditional role with some expanded or novel skills/ responsibilities which may have been jurisdiction of other professionals or disciplines	Funding may be short-term or pilot before substantive, based on results. Appointed by manager or lead of specialist team, which may not be a nurse (i.e. consultant pharmacist or physician in AMS)	Hospital or community	All, with mixture of planning, evaluation and delivery of services
Horizontal (i.e. staff nurse)	No	Limited or minimal strategic influence; most relations within own ward/team, with frequent contact with specialist/advisory roles (i.e. IPC specialists)	Feasible to attribute impact or clinical improvements in antimicrobial stewardship interventions deployed	Traditional role, supported by similar professionals in same or other clinicals areas	Mainstream human resources funding. Appointed by ward manager/nurse in charge	Hospital or community	Mainly clinical, educational, quality and managerial service delivery

### ‘Vertical’ model

Within this model, the participation of nurses in AMS efforts would be channelled via the implementation of a consultant nurse or advanced nurse practitioner role in AMS. The focus of this novel and often unique position would be AMS. In the UK, ‘consultant nurse’ roles were introduced 20 years ago structured around four core functions of expert practice of high autonomy; education, training and development; professional leadership to motivate others; service development, research and evaluation [27].

Due to these characteristics and aspirations, the roles may be ideal to transform organisational AMS practice. Beyond the creation or commissioning of the post, however, the overall structures for involvement and delivery of nursing input on stewardship would not be transformed at large and any clinical effect or improvement achieved stems from this expert, highly-specialised and influential individual [28].

The appointment of such figure would demonstrate organisational commitment and endorsement to improved AMS performance, and galvanise and rationalise efforts in AMS. Further, the introduction of this focal person in a position of decision-making and influence may legitimise the participation of nurses in AMS, in particular if the main remit of the role is designing, improving or leading practice. Without such guiding figure it may be challenging for nurses to engage in effective AMS, as seen in other areas such as infection prevention and control [29]. As this model would replicate organisational structures existing in other health professions involved in AMS decisions and management, integration within current clinical responsibilities and engagement of other professionals may be easier.

Potentially, though, generating hard evidence of positive outcomes or benefits achieved by the introduction of a single role or individual may be difficult. For example, consultant nurses have so far made their greatest impact in practice and service development and as knowledge brokers [30], rather than in direct patient outcomes and cost-benefit [31] thus being much more challenging to demonstrate such impact when any effect is achieved through the work of others. In antimicrobial decision-making, though, the emerging importance of behaviours such as persuading prescribers together with the social dynamics that end up shaping prescriptions has been gradually recognised [32], offering an ideal target for such advisory and influencing role. This also seems to have been the case with Consultant Pharmacists in Infection and AMR in the UK, encouraging the learning from previous experiences and across disciplines [33].

This hypothetical bottleneck in the translation from role to benefits could also hamper the scalability of the model and affect assumptions underpinning economic evaluations in view of the substantial start-up, upfront implementation costs required (i.e., setting up the role could be expensive) before realising any gains on AMS outcomes. Regarding expenditures, decision-makers may wish to account for any opportunity costs and avoid inefficiencies by reducing or eliminating potential overlapping areas with other professionals.

Finally, there could be a danger of reinforcing multi- but not inter-disciplinarity, by fostering parallel professional lines where a nurse, for example, would address and engage with nursing practices, whilst other professionals supervise and support their own cadres, in tune with existing evidence about social and team demarcations in antimicrobial prescribing [34]. To mitigate such fragmentation, a priori efforts would be vital to identify boundaries and relations between the spheres of responsibility and influence of the new role and others, for example between infection prevention and control, or specialist sepsis nurses [35].

### ‘Horizontal’ model

Within this approach there would not be any specific or dedicated AMS nurse role implemented. Instead, the organisation would engage in a concerted drive to increase optimal AMS behaviours and practices among nurses. Such behaviours may reflect nationally agreed objectives [36] (Table 4**)**, or conform to local needs.

**Table 4:** Components of ‘Start Smart then Focus’, [36]

- Do not start antibiotics in the absence of clinical evidence of bacterial infection.	
- For antibiotic(s) prescribed, document on drug chart and clinical notes: indication (including disease severity if appropriate), dose, route and duration or review date.	
- Obtain cultures first where possible.	
- Prescribe single dose antibiotics for surgical prophylaxis.	
- Review clinical diagnosis and continuing need for antibiotics by 48–72 h and make a clear plan of action - the ‘antimicrobial prescribing decision’.	
- The five ‘antimicrobial prescribing decision’ options are Stop, Switch, Change, Continue and OPAT.	
- It is essential that the review and subsequent decision be clearly documented in the clinical notes. The decision should also be documented clearly on the drug chart***.***	

This institutional perspective may serve to distance AMS from the point of prescription or decision-making about prescribing to a broader panoply of tasks, behaviours and decisions framed around the idea of providing excellent care. Doing so would address the recognised chasm between the awareness about AMS, a label hardly recognised by nurses [37], and many clinical tasks performed routinely – for example, ensuring that adequate and biological samples are sent on time for analysis, administering antibiotics doses on time, or communicating to the multidisciplinary team clinical improvements in the patient’s condition so alternative, less invasive therapy can be considered or stopped–, which account for most elements embedded in AMS and are central to quality care. Such point of view may also facilitate constructing a meaningful and engaging narrative for nurses, who may be much more receptive to messages framed around patient safety and quality than prescribing [38].

A great strength of this model would lie on its scalability and potential size and speed of its impact. For example, protocols and directives reflecting centrally-driven priorities about antimicrobial performance may be adopted with relative ease and therefore transform clinical practice without delay [39, 40]. In reality, such mandates tend to be met with organisational friction which undermines their effect, unless there is an adequate allocation of resources and responsibilities for one or several individuals in charge of the design, implementation, adoption and evaluation of these AMS tasks and responsibilities across the organisation and the nursing workforce [41] (Table 5).

**Table 5:** Examples of antimicrobial stewardship nursing posts from UK network

Domains							
Antimicrobial stewardship nursing posts*	Interprofessional working+	Strategic influence- Relation with other structures	Clinical outcomes- (What measure of impact? Process?)	Individual identity	Funding/ Managerial structures	Setting of practice (hospital, community…)	Role components (clinical, educational, quality, policy, managerial)
Nurse 1	Yes	Relation with infection prevention and control, pharmacy Evolving role focus on Carbapanemase-producing organism screening	Process	Staff Nurse	Infection Prevention & Sepsis Team, Nursing Directorate Substantive position	Hospital	Education
Nurse 2	Yes	Relation with infection prevention and control, pharmacy	Process	Staff nurse	Infection prevention and control, previously in pharmacy Substantive position	Hospital	Clinical, education
Nurse 3	Yes	Relation with infection prevention and control, pharmacy, antimicrobial stewardship team, university	Clinical outcomes, patient satisfaction, process	Advanced nurse practitioner	Infection Prevention and control/University Substantive position	Hospital	Clinical, education, policy, managerial
Nurse 4	Yes	Relation with infection prevention and control, antimicrobial stewardship team	Clinical outcomes, patient satisfaction, process	Lead nurse	Antimicrobial stewardship team Initial 1-year funding, then substantive position	Community and long-term care facilities	Clinical, education, policy
Nurse 5	Yes	Relation with infection prevention and control, antimicrobial stewardship team	Process	Staff nurse	Antimicrobial stewardship team. Substantive position	Hospital	Clinical
Nurse 6	Yes	Relation with infection prevention and control, and sepsis teams, but mainly on education for nurses	Clinical outcome, process	Staff nurse based within pharmacy	Antimicrobial stewardship team	Hospital	Clinical, quality improvement

*None of these roles exemplify the ‘horizontal’ approach theorised in the paper. +Equally, all roles explored work closely with other professions

In addition to the organisational friction, the horizontal model may have difficulties resolving competing tensions between existing paradigms within current antimicrobial management, with the response to sepsis demanding swift use of antibiotics whilst stewardship warrants a judicious, perhaps more measured, approach. Without a focal nursing figure establishing an integrative vision that recognises optimal use as the key behaviour (that is, prompt when needed yet promptly reviewed to determine whether its need remains relevant), nurses may not be able to engage in such nuanced performance.

### ‘Hybrid model’ (vertical-horizontal)

Within this model, organisations would foster and implement expectations about increased AMS tasks, roles, responsibilities embedded within the remit of all clinicians including nurses, underpinning such expectations with the allocation to existing healthcare workers of some expanded or specialist AMS skills. This approach could benefit from enhancing existing roles, such as infection prevention and control link nurses, or including further skills and responsibilities within existing specialist nursing teams – sepsis specialist nurses, or nurses in vascular access team would appear particularly well placed to embrace these additional responsibilities. Other posts such as ward managers could combine their leadership in infection prevention and control with an emphasis on stewardship.

This model would offer benefits due to the economies of scale derived from the use of a sizeable portion of the workforce, and natural links with existing roles. However, such rewards may stall due to a lack of clarity about a nurse-relevant vision to drive continued improvement, compounded by an increased complexity of the management of drug-resistant infections.

## Discussion

The broadening and expansion of nursing posts in AMS could strengthen existing programs [42]. However, the mere introduction of any roles –even if coupled with the development of associated educational competencies– may not be sufficient to stimulate benefits to patients, organisations and the wider healthcare economy.

A crucial aspect to realise these benefits would be the substantiation of clear, concrete, and precise markers of success for such nursing participation, ideally concerned about impact or outcomes rather than just process, in line with the current debate about optimal antimicrobial prescribing, for example [43, 44]. The challenges highlighted in the recent CDC/ANA White Paper [45] related to the existing absence of metrics that could reflect the benefit of incorporating nurses onto service delivery structures has to be acknowledged, and should encourage reflection at the local and national level, depending on the needs and resources available. Recent AMS experiences reported in rural and remote areas in Australia [46] validate the acute need to tailor interventions to the local context.

Although comparable analyses of AMS nursing models in the UK or elsewhere are lacking, it would be possible to apply our findings to some of the existing literature describing the relation of nurses with existing AMS interventions. For example, in their survey exploring the participation of nurses in stewardship across the African continent Bulabula et al. (2018) [47] advocate for expanding the role of clinical nurses to support stewardship, particularly in rural areas, an idea that aligns completely with the horizontal model of wide addition of skills and knowledge to the existing workforce. Further, these authors recognise how existing IPC nurses may also already attend AMS committees and input on policy and clinical decisions, a scenario fitting to our hybrid model of practice. Another example of such hybrid practice appears in a qualitative study conducted in the US [48] documenting the engagement of nurses in optimising the use of antibiotics in intensive care units. The authors propose that existing advanced nurse practitioners embrace AMS leadership roles, so the involvement of other nursing colleagues is galvanized and sustained.

Within the UK network the appointment of single professionals on short, fixed-term contracts could be of concern and raise doubts about the likelihood of achieving meaningful clinical and organisational impacts. Such short-term appointing may reflect the business reality of attempting to create new positions. However, as the evidence mounts of benefits related to the participation of nurses, the focus should instead be placed on how best implement new roles or responsibilities rather than, in effect, whether to deploy them.

Additionally, decision-makers evaluating whether to fund and support nurse roles in AMS ought to elude potential unintended consequences derived from such appointments. For example, it would be paramount for AMS not be seen as an isolated, specialist issue that is better handled by specialists, therefore leading to the de-skilling [49] or disinterest from the generalist nursing workforce. As seen in medical practice [50], non-specialist practitioners are still greatly involved in, and remain responsible, for the majority of AMR-related actions.

In fact, the existing narrative and experiences about AMS nursing have mainly involved secondary care, with a handful of experiences in community and long-term facilities [51]. Considering that the largest proportion of antibiotics are prescribed in primary care and that nurses are responsible for delivering most services, such imbalance requires urgent attention. The current political emphasis on universal health coverage supported by robust nursing leadership may offer an optimal prospect for studies of community stewardship roles.

## Conclusion

In summary, pressing clinical demands imposed by antimicrobial resistance require organisational responses that would be remiss to ignore the contribution that nurses can have. Optimal approaches to incorporate such contribution remain to be agreed upon, in particular the executive and leadership rather than the clinical facets of the nurse participation in AMS. Although solutions should be context sensitive, resource dependent and pragmatic, the horizontal model of AMS nursing may allow for rapid system-level engagement and linkage to existing structures such as AMS pharmacist, which would facilitate mutual support in achieving AMS goals.

## Data Availability

Not applicable.

## Abbreviations

AMS: Antimicrobial stewardship
ANA: American Nurses Association
CDC: Centers for Disease Control and Prevention
DRI: Drug-resistant infections
SSTF: Start Smart Then Focus
UK: United Kingdom

## References

[1] Dyar OJ, Huttner B, Schouten J (2017). Pulcini C; ESGAP (ESCMID study Group for Antimicrobial stewardshiP). What is antimicrobial stewardship?. Clin Microbiol Infect.

[2] The Review on Antimicrobial Resistance Final report. [Accessed at http://amr-review.org/Publications]. Accessed 11 May 2019.

[3] Campbell J, Dussault G, Buchan J, Pozo-Martin F, Guerra Arias M, Leone C, Siyam A, Cometto G (2013). A universal truth: no health without a workforce. Forum Report, Third Global Forum on Human Resources for Health.

[4] World Health Organization. World Health Statistics 2011. Geneva: World Health Organization; 2012. Accessed at https://www.who.int/whosis/whostat/EN_WHS2011_Full.pdf?ua=1.

[5] Total Number of Professionally Active Nurses. [Accessed at https://www.kff.org/other/state-indicator/total-registered-nurses/?currentTimeframe=0&sortModel=%7B%22colId%22:%22Location%22,%22sort%22:%22asc%22%7D]. Accessed 23 May 2019.

[6] NHS Statistics, Facts, and Figures. [Accessed at https://www.nhsconfed.org/resources/key-statistics-on-the-nhs]. Accessed 05 May 2019.

[7] Olans RN, Olans RD, DeMaria A (2016). The critical role of the staff nurse in antimicrobial stewardship: unrecognized, but already there. Clin Infect Dis.

[8] Olans RD, Olans RN, Witt D (2017). Good nursing is good antibiotic stewardship. Am J Nurs.

[9] Edwards R, Drumright LN, Kiernan M, Holmes A (2011). Covering more territory to fight resistance: considering nurses' role in antimicrobial stewardship. J Infect Prev.

[10] Castro-Sánchez E, Bennasar-Veny M, Smith M, Singleton S, Bennett E, Appleton J (2018). European Commission guidelines for the prudent use of antimicrobials in human health: a missed opportunity to embrace nursing participation in stewardship. Clin Microb Infect.

[11] Castro-Sánchez E, Drumright LN, Gharbi M, Farrell S, Holmes AH (2016). Mapping antimicrobial stewardship in undergraduate medical, dental, pharmacy, nursing and veterinary education in the United Kingdom. PLoS One.

[12] Rawson TM, Butters TP, Moore LS, Castro-Sánchez E, Cooke FJ, Holmes AH (2016). Exploring the coverage of antimicrobial stewardship across UK clinical postgraduate training curricula. J Antimicrob Chemother.

[13] Courtenay M, Lim R, Castro-Sanchez E, Deslandes R, Hodson K, Morris G (2018). Development of consensus-based national antimicrobial stewardship competencies for UK undergraduate healthcare professional education. J Hosp Infect.

[14] Dyar O.J., Beović B., Pulcini C., Tacconelli E., Hulscher M., Cookson B., Ashiru-Oredope D., Barcs I., Blix H.S., Buyle F., Chowers M., Čižman M., Cookson B., Del Pozo J.L., Deptula A., Dumpis U., Florea D., van de Garde E., Geffen Y., Giske C.G., Grau S., Hajdú E., Hell M., Hondo Ł., Hussein K., Huttner B., Kern W., Kernéis S., Knepper V., Kofteridis D., Kostyanev T., Kuijper E., Lebanova H., Lewis R., Cordina C.M., Matulionyte R., Maurer F., Messiaen P., Miciuleviciene J., Mrhar A., Nabuurs-Franssen M., Naesens R., Oxacelay C., Pagani L., Paño-Pardo J.R., Paul M., Petrikkos G., Pluess-Suard C., Popescu G.A., Porsche U., Prins J., Pulcini C., Rello J., Rodríguez-Baño J., Rossolini G.M., Salzberger B., Seme K., Simonsen G.S., Sînziana M., Skovgaard S., Smith I., Sönsken U., Soriano A., Sviestiņa I., Szilagyi E., Tängdén T., Tattevin P., Tsioutis C., Vilde A., Wanke-Rytt M., Wechsler-Fördös A., Zarb P. (2019). ESCMID generic competencies in antimicrobial prescribing and stewardship: towards a European consensus. Clinical Microbiology and Infection.

[15] WHO (2018). Competency framework for health workers’ education and training on antimicrobial resistance.

[16] Pulcini C., Binda F., Lamkang A.S., Trett A., Charani E., Goff D.A., Harbarth S., Hinrichsen S.L., Levy-Hara G., Mendelson M., Nathwani D., Gunturu R., Singh S., Srinivasan A., Thamlikitkul V., Thursky K., Vlieghe E., Wertheim H., Zeng M., Gandra S., Laxminarayan R. (2019). Developing core elements and checklist items for global hospital antimicrobial stewardship programmes: a consensus approach. Clinical Microbiology and Infection.

[17] Manning ML, Gianuzzi D (2015). Keeping patients safe: antibiotic resistance and the role of nurse executives in antibiotic stewardship. J Nurs Admin.

[18] Carlet J, Pulcini C, Piddock LJ (2014). Antibiotic resistance: a geopolitical issue. Clin Microbiol Infect.

[19] Castro-Sánchez E, Gilchrist MJ, McEwen J, Smith M, Kennedy H, Holmes A (2017). Antimicrobial stewardship: widening the collaborative approach. J Antimicrob Stewardship.

[20] Ranmuthugala G, Plumb JJ, Cunningham FC, Georgiou A, Westbrook JI, Braithwaite J (2011). How and why are communities of practice established in the healthcare sector? A systematic review of the literature. BMC Health Serv Res.

[21] International Council of Nurses. Position Statement on Antimicrobial Resistance. 2009 [Accessed at https://www.icn.ch/sites/default/files/inline-files/ICN_PS_Antimicrobial_resistance.pdf]. Accessed 21 Apr 2019.

[22] Barlam Tamar F., Cosgrove Sara E., Abbo Lilian M., MacDougall Conan, Schuetz Audrey N., Septimus Edward J., Srinivasan Arjun, Dellit Timothy H., Falck-Ytter Yngve T., Fishman Neil O., Hamilton Cindy W., Jenkins Timothy C., Lipsett Pamela A., Malani Preeti N., May Larissa S., Moran Gregory J., Neuhauser Melinda M., Newland Jason G., Ohl Christopher A., Samore Matthew H., Seo Susan K., Trivedi Kavita K. (2016). Implementing an Antibiotic Stewardship Program: Guidelines by the Infectious Diseases Society of America and the Society for Healthcare Epidemiology of America. Clinical Infectious Diseases.

[23] Fairall L, Bachmann MO, Lombard C, Timmerman V, Uebel K, Zwarenstein M (2012). Task shifting of antiretroviral treatment from doctors to primary-care nurses in South Africa (STRETCH): a pragmatic, parallel, cluster-randomised trial. Lancet.

[24] Colvin CJ, Fairall L, Lewin S, Georgeu D, Zwarenstein M, Bachmann MO (2010). Expanding access to ART in South Africa: the role of nurse-initiated treatment. S Afr Med J.

[25] Georgeu D, Colvin CJ, Lewin S, Fairall L, Bachmann MO, Uebel K (2012). Implementing nurse-initiated and managed antiretroviral treatment (NIMART) in South Africa: a qualitative process evaluation of the STRETCH trial. Implement Sci.

[26] Kredo T, Adeniyi FB, Bateganya M, Pienaar ED (2014). Task shifting from doctors to non-doctors for initiation and maintenance of antiretroviral therapy. Cochrane Database Syst Rev.

[27] Department of Health (1999). Making a Difference: Strengthening the nursing, midwifery and health visiting contribution to health care.

[28] Humphreys A, Johnson S, Richardson J, Stenhouse E, Watkins M (2007). A systematic review and meta-synthesis: evaluating the effectiveness of nurse, midwife/allied health professional consultants. J Clin Nursing.

[29] Barnes Sue, Zirges Chris, Tomac Dawn, Hall-Meyer Kathleen, Stein Linda, Barnden Marsha, Studer Marsha, Bowers Tim, Brinsko Vicki (2019). The emerging role of the corporate or system-level infection prevention director for integrated delivery networks. American Journal of Infection Control.

[30] Giles M, Parker V, Conway J, Mitchell R (2018). Knowing how to get things done: nurse consultants as clinical leaders. J Clin Nurs.

[31] Franks H, Howarth M (2012). Being an effective nurse consultant in the English National Health Service: what does it take? A study of consultants specializing in safeguarding. J Nurs Manag.

[32] Charani E, Castro-Sanchez E, Sevdalis N, Kyratsis Y, Drumright L, Shah N (2013). Understanding the determinants of antimicrobial prescribing within hospitals: the role of "prescribing etiquette". Clin Infect Dis.

[33] Gilchrist M, Wade P, Ashiru-Oredope D, Howard P, Sneddon J, Whitney L (2015). Antimicrobial stewardship from policy to practice: Experiences from UK antimicrobial pharmacists. Infect Dis Ther.

[34] Broom A, Broom J, Kirby E, Scambler G (2015). The path of least resistance? Jurisdictions, responsibility and professional asymmetries in pharmacists' accounts of antibiotic decisions in hospitals. Soc Sci Med.

[35] Steffens Ester, Quintens Charlotte, Derdelinckx Inge, Peetermans Willy E., Van Eldere Johan, Spriet Isabel, Schuermans Annette (2018). Outpatient parenteral antimicrobial therapy and antibiotic stewardship: opponents or teammates?. Infection.

[36] Ashiru-Oredope D, Sharland M, Charani E, McNulty C (2012). Cooke J; ARHAI antimicrobial stewardship group. Improving the quality of antibiotic prescribing in the NHS by developing a new antimicrobial stewardship Programme: start smart--then focus. J Antimicrob Chemother.

[37] Greendyke WG, Carter EJ, Salsgiver E, Bernstein D, Simon MS, Saiman L (2018). Exploring the role of the bedside nurse in antimicrobial stewardship: survey results from five acute-care hospitals. Infect Control Hosp Epidemiol.

[38] Grayson ML, Macesic N, Huang GK, Bond K, Fletcher J, Gilbert GL (2015). Use of an innovative personality-mindset profiling tool to guide culture-change strategies among different healthcare worker groups. PLoS One.

[39] Romero B, Fry M, Roche M (2017). The impact of evidence-based sepsis guidelines on emergency department clinical practice: a pre-post medical record audit. J Clin Nurs.

[40] Ouldali N, Bellêttre X, Milcent K, Guedj R, de Pontual L, Cojocaru B (2017). Impact of implementing National Guidelines on antibiotic prescriptions for acute respiratory tract infections in pediatric emergency departments: an interrupted time series analysis. Clin Infect Dis.

[41] Kirby E, Broom A, Gibson A, Broom J, Yarwood T, Post J (2018). Medical authority, managerial power and political will: a Bourdieusian analysis of antibiotics in the hospital. Health (London).

[42] Ha David R., Forte Mary Bette, Olans Rita D., OYong Kelsey, Olans Richard N., Gluckstein Daniel P., Kullar Ravina, Desai Mamta, Catipon Nora, Ancheta Vickie, Lira Donna, Khattak Yesenia, Legge Jessica, Nguyen Kim B., Chan Sarah, Mourani John, McKinnell James A. (2019). A Multidisciplinary Approach to Incorporate Bedside Nurses into Antimicrobial Stewardship and Infection Prevention. The Joint Commission Journal on Quality and Patient Safety.

[43] Stanic Benic M, Milanic R, Monnier AA (2018). Metrics for quantifying antibiotic use in the hospital setting: results from a systematic review and international multidisciplinary consensus procedure. J Antimicrob Chemother.

[44] Versporten A, Gyssens IC, Pulcini C, Monnier AA, Schouten J, Milanič R (2018). Metrics to assess the quantity of antibiotic use in the outpatient setting: a systematic review followed by an international multidisciplinary consensus procedure. J Antimicrob Chemother.

[45] ANA & CDC (2017). Redefining the Antibiotic Stewardship Team: Recommendations from the American Nurses Association/Centers for Disease Control and Prevention Workgroup on the Role of Registered Nurses in Hospital Antibiotic Stewardship Practices.

[46] Bishop J, Kong DC, Schulz TR, Thursky KA, Buising KL (2018). Meeting the challenge for effective antimicrobial stewardship programs in regional, rural and remote hospitals - what can we learn from the published literature?. Rural Remote Health.

[47] Bulabula ANH, Jenkins A, Mehtar S, Nathwani D (2018). Education and management of antimicrobials amongst nurses in Africa-a situation analysis: an infection control Africa network (ICAN)/BSAC online survey. J Antimicrob Chemother.

[48] Jeffs L, Law MP, Zahradnik M, Steinberg M, Maione M, Jorgoni L, Bell CM, Morris AM (2018). Engaging nurses in optimizing antimicrobial use in ICUs: a qualitative study. J Nurs Care Qual.

[49] Gorman SK, Pro RS, Dresser LD, Con PE (2016). Should Traditional Antimicrobial Stewardship (AMS) Models Incorporating Clinical Pharmacists with Full-Time AMS Responsibilities Be Replaced by Models in Which Pharmacists Simply Participate in AMS Activities as Part of Their Routine Ward or Team-Based Pharmaceutical Care?. Can J Hosp Pharm.

[50] Rawson TM, Moore LSP, Gilchrist MJ, Holmes AH (2016). Antimicrobial stewardship: are we failing in cross-specialty clinical engagement?. J Antimicrob Chemother.

[51] CDC (2015). The Core Elements of Antibiotic Stewardship for Nursing Homes.

